# Successful Use of Argatroban to Treat a Critically Ill Patient with Coagulopathy and Nephropathy Secondary to COVID-19

**DOI:** 10.1055/s-0040-1721501

**Published:** 2020-12-15

**Authors:** Matthew C. Frise, Rebecca E.V. Gates, Nicola S. Curry, Christopher M. Danbury

**Affiliations:** 1 Royal Berkshire NHS Foundation Trust, Royal Berkshire Hospital, Reading, United Kingdom; 2 Department of Physiology, Anatomy and Genetics, University of Oxford, Oxford, United Kingdom; 3 Oxford University Hospitals NHS Foundation Trust, Churchill Hospital, Headington, Oxford, United Kingdom

Data from early case series of patients infected with severe acute respiratory syndrome coronavirus 2 (SARS-CoV-2) indicated that disordered thrombosis and hemostasis were frequent features of coronavirus disease 2019 (COVID-19) portending a worse outcome. 1 2 3 4 For example, in a case series of 191 patients from two Wuhan hospitals, an elevated admission D-dimer level was associated with almost a 20-fold increased risk of death. 5 A further single center study of 183 patients from another Wuhan hospital found that elevated fibrin degradation product levels, as well as a prolonged prothrombin time and activated partial thromboplastin time (APTT), were associated with a significantly higher mortality. 6

As the pandemic has progressed, it has become clear that while the coagulopathy of COVID-19 shares features with both disseminated intravascular coagulation (DIC) 7 and thrombotic microangiopathy (TMA), 8 several aspects make it distinct from each of these entities. 9 10 It has even been suggested that in an extreme form, the coagulopathy of COVID-19 resembles antiphospholipid syndrome, evidenced by multiple thrombotic events and positive serological tests in keeping with such a diagnosis. 11 12

One aspect of the challenges posed by this unusual coagulopathy that has received relatively little attention is the impact on instituting renal replacement therapy (RRT). Acute kidney injury necessitating RRT occurs in at least one in five COVID-19 patients requiring intensive care unit (ICU) admission. 13 14 Difficulties related to thrombosis of extracorporeal circuits have been described 15 16 ; strategies to overcome this problem are urgently required, not simply so that RRT can be instituted in a timely manner and delivered effectively, but also because prolonging circuit life is essential in the context of a pandemic when supplies of consumables risk becoming exhausted. 14

Here we describe a patient with multiple organ failure secondary to COVID-19 who exhibited significant derangement of laboratory coagulation indices, and in whom attempts to institute acute RRT were hampered by repeated thrombosis of the hemofiltration circuit and vascular access catheters. In late March 2020, the patient, a 62-year-old man with hypertension, obesity, and hypercholesterolemia, presented to the Emergency Department with profound hypoxemia refractory to supplemental oxygen therapy. Admission laboratory findings of note included a D-dimer of 1,868 µg FEU/L, fibrinogen 10.17 g/L, creatinine 150 µmol/L, urea 12.2 mmol/L, C-reactive protein 364 mg/L, and ferritin 2,428 ug/L. He required intubation, mechanical ventilation, and circulatory support with norepinephrine, and went on to become anuric within 24 hours.

Ten hours after admission the decision was taken to institute continuous venovenous hemodiafiltration (CVVHDF). A 12 Fr 20-cm dual-lumen Arrow vascular access catheter (Teleflex Inc, Reading, Pennsylvania, United States) was inserted into the right femoral vein under real-time ultrasound guidance. CVVHDF was commenced using a Prismaflex platform and ST150 filter set (Baxter International Inc, Deerfield, Illinois, United States) at 25 mL/kg/h using a predilution strategy. Anticoagulation was provided by systemic infusion of unfractionated heparin (UFH) aiming for an APTT of 45 to 70 seconds. It was impossible to institute effective RRT, as evidenced by a relentlessly climbing serum creatinine despite multiple attempts and repeated insertion of new vascular access catheters at different sites. The hemofiltration circuits clotted very quickly, one of them within minutes of commencement of therapy. Tinzaparin 175 units/kg once daily was given on three occasions in addition to intravenous UFH, with no obvious benefit. Fig. 1 illustrates these events.

Six days into his ICU stay all heparin was discontinued and an infusion of argatroban commenced at an initial rate of 0.5 µg/kg/min. The APTT was measured every 4 hours and the infusion rate increased by 0.1 µg/kg/min until the APTT ratio was consistently between 1.5 and 3. A rapid improvement in the life of the hemofiltration circuits and vascular access catheters followed, and a corresponding decrease in the serum creatinine was swiftly observed. After several days of argatroban therapy, a tunneled semi-permanent dialysis catheter was sited under fluoroscopic guidance. This continued to function throughout a subsequent tracheostomy wean. On the 18th day of his admission the D-dimer had risen to 6,309 µg FEU/L, ultimately peaking at 18,775 µg FEU/L on the 28th day. He was eventually successfully decannulated, became independent of RRT, and was discharged home after nearly 2 months in hospital.

Many intensivists will be familiar with the utility of argatroban, a small molecule direct thrombin inhibitor, from its use in critically ill patients with heparin-induced thrombocytopenia. 17 That we overcame the difficulties encountered in this case using argatroban supports the view that heparin resistance plays a central role in the coagulopathy of COVID-19. It also accords with the observation that rates of venous thromboembolism (VTE) in critically ill patients with COVID-19 are extremely high when prophylactic anticoagulation with low molecular weight heparin is used, and that VTE can be detected in the majority of patients with severe COVID-19 even when therapeutic anticoagulation is employed empirically early in the course of the disease. 18 Heparin resistance in this setting has been attributed to the combination of high factor VIII and fibrinogen levels accompanied by low circulating antithrombin, 19 a picture that has been described as a prothrombotic variant of DIC. 20

Since our patient's discharge from hospital, a single-center study has suggested argatroban to be effective when utilized as an escalation therapy in patients with COVID-19 who have already developed thromboembolism and who require continuous RRT. 21 Additionally, argatroban use has been described in a small group of patients more profoundly critically ill with SARS-CoV-2 infection, most of whom required extracorporeal membrane oxygenation (ECMO). 22 Aside from RRT and ECMO, an extracorporeal approach to removing circulating SARS-CoV-2 from the bloodstream is also being explored 23 ; effective anticoagulation strategies will be essential if such extracorporeal therapies are to be successful.

Further studies are urgently required to determine the molecular pathogenesis of, and optimum treatment for, the coagulopathy seen in COVID-19. 3 4 Although direct infection of renal tissue by SARS-CoV-2 appears to be of greater importance than TMA in the pathophysiology of the nephropathy seen in the condition, 24 the prothrombotic state may seriously compromise the supportive care of these patients when critically ill. We believe it is important to highlight our experience with argatroban in this patient, in whom it appeared to be life-saving. We believe that the efficacy of argatroban in this setting should be explored in a randomized controlled trial.

**Fig. 1:**
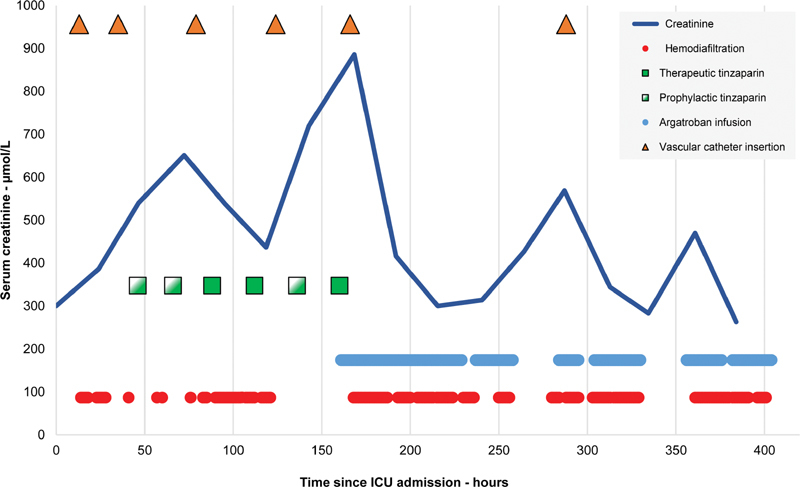
Anticoagulation therapy, vascular access catheter insertions, and periods of hemodiafiltration after ICU admission. The final line inserted was a tunneled semi-permanent dialysis catheter. ICU, intensive care unit.

## References

[1] Lippi G Favaloro E J D-dimer is associated with severity of coronavirus disease 2019: a pooled analysis Thromb Haemost 2020 120 05 876 878 32246450 10.1055/s-0040-1709650 PMC7295300

[2] Terpos E Ntanasis-Stathopoulos I Elalamy I Hematological findings and complications of COVID-19 Am J Hematol 2020 95 07 834 847 32282949 10.1002/ajh.25829 PMC7262337

[3] Kollias A Kyriakoulis K G Dimakakos E Poulakou G Stergiou G S Syrigos K Thromboembolic risk and anticoagulant therapy in COVID-19 patients: emerging evidence and call for action Br J Haematol 2020 189 05 846 847 32304577 10.1111/bjh.16727 PMC7264537

[4] Levi M Thachil J Iba T Levy J H Coagulation abnormalities and thrombosis in patients with COVID-19 Lancet Haematol 2020 7 06 e438 e440 32407672 10.1016/S2352-3026(20)30145-9 PMC7213964

[5] Zhou F Yu T Du R Clinical course and risk factors for mortality of adult inpatients with COVID-19 in Wuhan, China: a retrospective cohort study Lancet 2020 395(10229):1054 1062 32171076 10.1016/S0140-6736(20)30566-3 PMC7270627

[6] Tang N Li D Wang X Sun Z Abnormal coagulation parameters are associated with poor prognosis in patients with novel coronavirus pneumonia J Thromb Haemost 2020 18 04 844 847 32073213 10.1111/jth.14768 PMC7166509

[7] Violi F Pastori D Cangemi R Pignatelli P Loffredo L Hypercoagulation and antithrombotic treatment in coronavirus 2019: a new challenge Thromb Haemost 2020 120 06 949 956 32349133 10.1055/s-0040-1710317 PMC7295290

[8] Global COVID-19 Thrombosis Collaborative Group Bikdeli B Madhavan M V Gupta A Pharmacological agents targeting thromboinflammation in COVID-19: review and implications for future research Thromb Haemost 2020 120 07 1004 1024 32473596 10.1055/s-0040-1713152 PMC7516364

[9] Iba T Levy J H Levi M Connors J M Thachil J Coagulopathy of coronavirus disease 2019 Crit Care Med 2020 48 09 1358 1364 32467443 10.1097/CCM.0000000000004458 PMC7255402

[10] Spiezia L Boscolo A Poletto F COVID-19-related severe hypercoagulability in patients admitted to intensive care unit for acute respiratory failure Thromb Haemost 2020 120 06 998 1000 32316063 10.1055/s-0040-1710018 PMC7295272

[11] Zhang Y Xiao M Zhang S Coagulopathy and antiphospholipid antibodies in patients with COVID-19 N Engl J Med 2020 382 17 e38 32268022 10.1056/NEJMc2007575 PMC7161262

[12] Harzallah I Debliquis A Drénou B Lupus anticoagulant is frequent in patients with COVID-19 J Thromb Haemost 2020 18 08 2064 2065 32324958 10.1111/jth.14867 PMC7264773

[13] Ronco C Reis T Husain-Syed F Management of acute kidney injury in patients with COVID-19 Lancet Respir Med 2020 8 07 738 742 32416769 10.1016/S2213-2600(20)30229-0 PMC7255232

[14] Goldfarb D S Benstein J A Zhdanova O Impending shortages of kidney replacement therapy for COVID-19 patients Clin J Am Soc Nephrol 2020 15 06 880 882 32345750 10.2215/CJN.05180420 PMC7274293

[15] Sise M E Baggett M V Shepard J O Stevens J S Rhee E P Case 17-2020: A 68-year-old man with COVID-19 and acute kidney injury N Engl J Med 2020 382 22 2147 2156 32402156 10.1056/NEJMcpc2002418 PMC7959270

[16] Wilbers T J Koning M V Renal replacement therapy in critically ill patients with COVID-19: a retrospective study investigating mortality, renal recovery and filter lifetime J Crit Care 2020 60 103 105 32795841 10.1016/j.jcrc.2020.07.025 PMC7391167

[17] Saugel B Phillip V Moessmer G Schmid R M Huber W Argatroban therapy for heparin-induced thrombocytopenia in ICU patients with multiple organ dysfunction syndrome: a retrospective study Crit Care 2010 14 03 R90 20487559 10.1186/cc9024 PMC2911727

[18] Llitjos J F Leclerc M Chochois C High incidence of venous thromboembolic events in anticoagulated severe COVID-19 patients J Thromb Haemost 2020 18 07 1743 1746 32320517 10.1111/jth.14869 PMC7264774

[19] White D MacDonald S Bull T Heparin resistance in COVID-19 patients in the intensive care unit J Thromb Thrombolysis 2020 50 02 287 291 32445064 10.1007/s11239-020-02145-0 PMC7242778

[20] Wang J Hajizadeh N Moore E E Tissue plasminogen activator (tPA) treatment for COVID-19 associated acute respiratory distress syndrome (ARDS): a case series J Thromb Haemost 2020 18 07 1752 1755 32267998 10.1111/jth.14828 PMC7262152

[21] Shankaranarayanan D Muthukumar T Barbar T Anticoagulation strategies and filter life in COVID-19 patients receiving continuous renal replacement therapy: a single-center experience Clin J Am Soc Nephrol 2020(e-pub ahead of print) 10.2215/CJN.08430520 PMC7792651 32943397

[22] Arachchillage D J Remmington C Rosenberg A Anticoagulation with argatroban in patients with acute antithrombin deficiency in severe COVID-19 Br J Haematol 2020 190 05 e286 e288 32516429 10.1111/bjh.16927 PMC7300519

[23] Seffer M T Cottam D Forni L G Kielstein J T Heparin 2.0: a new approach to the infection crisis Blood Purif 2020(e-pub ahead of print) 10.1159/000508647 PMC7445380 32615569

[24] Su H Yang M Wan C Renal histopathological analysis of 26 postmortem findings of patients with COVID-19 in China Kidney Int 2020 98 01 219 227 32327202 10.1016/j.kint.2020.04.003 PMC7194105

